# What Is Cincho-Quinine?

**Published:** 1873

**Authors:** 


### WHAT IS CINCHO-QUININE?

This question is often asked by physicians who have not been made acquainted with the nature of this important agent, and therefore we republish the following article, which appeared in the Journal in June, 1869, and which presents in a clear and explicit manner its nature and uses:

The chemical manipulation of the Cinchona or Peruvian barks reveals the presence in them of quite a number of most remarkable complex bodies. No vegetable production, except the poppy, affords such a marvelous combination of valuable medicinal principles as the *loxa* and *calisaya* barks, and no substances have been studied with greater care or more intense interest by chemists. Nothing short of the subtle chemical forces controlled by the Infinite One could construct from the elements of the earth and air a bitter principle like quinia, or those agents associated in bark, so closely allied to it physically and chemically. A handful of the finely comminuted fibres of the yellow bark, which resembles physically a dozen other varieties, is made to yield by the chemist, when treated with aqueous and alcoholic liquids, and acids, a dark, bitter solution, unattractive in taste and appearance. If the process is skillfully conducted, or exhaustive in its results, there remains, besides the solution, a portion of woody fibre, inert and almost tasteless. It holds considerable coloring and some waxy matter, together with a little tannin; but the active chemical or medicinal principles have been removed, and are held in the dark liquid. The exhausted bark is not entirely worthless, for it may be dried and used as fuel. But what of the dark liquid? From this the chemist obtains, besides other substances, a portion of beautiful white, silky crystals; not wholly of one distinct kind, but of several, all of which possess about equal chemical and therapeutical importance. No wonder it seems to the uninitiated in chemical manipulation a difficult work to perform. It is, however, quite easy to the thoroughly instructed. The first principle isolated may be the quinia. This is not held in the bark in its naked alkaloidal condition, but locked up, in the form of a salt, with another principle called *kinic acid.* In the bark it is *kinate of quinia.* We isolate the quinia, tear it from its embrace with kinic acid, throw that away, and force it into a kind of matrimonial alliance with sulphuric acid, and in this condition of *sulphate of quinia*, use it as a medicine. This kinic acid marries into several other families resident in the bark, prominent among which are *cinchonia*, *cinchonidia*, *quinidia*, etc. Precisely how many of these alkaloidal principles the different kinds of barks contain, is unknown; but it is safe to assume that there are as many as four others which, although not distinctly pointed out, are tolerably well recognized. These *kinates* are all *kindred* in nature, and all labor to the same end, when isolated and set to work as therapeutical agents in the human system.

In one hundred ounces of good yellow bark, we obtain about two and three-fourths ounces of quinia, and two ounces of cinchonia, with variable amounts of the other principles, but less than the two named. It is to be regretted that we cannot remove the different families of kinates from the bark in their natural state of saline combination. It seems reasonable to suppose their action upon the system would be more salutary than in other forms. It is easy to isolate the kinic acid, and having the alkaloids, the kinates of quinia, cinchonia, etc., can be re-formed; but in these chemical changes so much disturbance to natural organic combinations is made, that practically, we realize no marked advantages. It seems unnatural to force a natural alkaloidal base out of its association with an organic acid, and recombine it with mineral acid. This we do in the preparation of the sulphate of quinia. However, as it has served so good a purpose for many years, it is not best to quarrel with theory.

All the alkaloids of bark possess about equal febrifuge and tonic properties, when isolated and administered in that condition. This has been proved over and over again by all competent chemists and physicians, from Drs. Gomez, Duncan, Pelletier, Caventou, down to the time of Liebig's researches, a quarter of a century ago, and from that time to the present by a hundred careful chemical and medical observers.

How the one alkaloid, quinia, came to supersede the others, and drive them into the background, is easily understood, when we remember that it was about the first that was distinctly eliminated, studied, and experimented with; and the *eclat* it acquired caused everything else to be neglected. The natural bark, holding all the alkaloids, the quinia, cinchonia, quinidia, etc., has always been observed to produce more efficient and prompt results, both as a tonic and febrifuge, than the quinia, or either of the other principles in themselves: but holding also, as it does, tannin, gum, starch, fibrine, and coloring matter, all of which are medicinally interfering or inert, its use is rendered inconvenient and inadmissable in many cases. Besides, it is apt to produce disturbance of the gastric functions of an unpleasant character. Acting upon the idea that the unnatural alkaloidal principles of bark, in their simple, unchanged condition, separated from the gross, woody, and other matters, would better subserve all therapeutical ends than the barks themselves, or *any one* of the alkaloids separately employed, Cincho-Quinine has been prepared.

Cincho-Quinine contains no external agents, as sugar, licorice, starch, magnesia, etc. *It is wholly composed of the bark alkaloids:* 1st, quinia; 2d, cinchonia; 3d, quinidia; 4th, cinchonidia; 5th, other alkaloidal principles present in barks, which have not been distinctly isolated, and the precise nature of which are not well understood. In the beautiful white amorphus scales of Chinco-Quinine, the whole of the active febrifuge and tonic principles of the cinchonia barks are secured without the inert, bulky lignin, gum, etc. It is believed to have these advantages over sulphate of quinine:

1st. It exerts the full therapeutic influence of quinine, in the same doses, without oppressing the stomach or creating nausea. It does not produce cerebral distress, as sulphate of quinine is apt to do, and in the large number of cases in which it has been tried, it has been found to produce much less constitutional disturbance.

2d. *It has the great advantage of being nearly tasteless.* The bitter is very slight, and not unpleasant to the most sensitive, delicate woman or child.

3d. It is less costly than sulphate of quinine. Like the sulphate of quinine, the price will fluctuate with the rise and fall of barks, but it will always be less than the lowest market price of that salt.

4th. It meets indications not met by that salt.

